# Use of sustained release dextroamphetamine for the treatment of stimulant use disorder in the setting of injectable opioid agonist treatment in Canada: a case report

**DOI:** 10.1186/s12954-021-00500-9

**Published:** 2021-05-20

**Authors:** Heather Palis, Scott MacDonald, Jennifer Jun, Eugenia Oviedo-Joekes

**Affiliations:** 1 Centre for Health Evaluation and Outcome Sciences, Providence Health Care, St. Pauls Hospital, 575-1081 Burrard St., Vancouver, BC V6Z 1Y6 Canada; 2 Providence Health Care, Providence Crosstown Clinic, 84 West Hastings Street, Vancouver, BC V6B 1G6 Canada; 3 School of Population and Public Health, University of British Columbia, 2206 East Mall, , Vancouver, BC V6T 1Z3 Canada

**Keywords:** Case report, Stimulant use disorder, Opioid use disorder, Pharmacological treatment, Dextroamphetamine, Cocaine

## Abstract

**Background:**

For people with opioid use disorder who are not responding to oral opioid agonist treatment, evidence supports the effectiveness of injectable opioid agonist treatment with injectable hydromorphone (an opioid analgesic) and diacetylmorphine (pharmaceutical grade heroin). While this treatment is effective at reducing illicit opioid use, concurrent cocaine use is prevalent. Dextroamphetamine (a central nervous system stimulant) has been found to be a safe and effective treatment for cocaine dependence among people receiving injectable opioid agonist treatment in Europe. We present the first report of dextroamphetamine prescribing offered for the treatment of stimulant use disorder among a patient receiving iOAT outside of a clinical trial. This case report can be used to inform clinical practice in the treatment of cocaine use disorder, an area where interventions are currently lacking.

**Case presentation:**

Dextroamphetamine was prescribed to a 51-year-old male who was diagnosed with concurrent opioid and stimulant use disorder in an injectable opioid agonist treatment clinic in Vancouver, Canada. He reported smoking crack cocaine daily for more than two decades and was experiencing health consequences associated with this use. He presented to his routine physician visit with the goal of reducing his cocaine use and was prescribed dextroamphetamine for the treatment of stimulant use disorder. After 4-weeks the patient was tolerating the medication with no observed adverse events and was achieving his therapeutic goal of reducing his cocaine use.

**Conclusions:**

Dextroamphetamine can be prescribed to support patients with stimulant use disorder to reduce or stop their use of cocaine. The case demonstrated that when dextroamphetamine was prescribed, a significant reduction in cocaine use was experienced among a patient that had been regularly using cocaine on a daily basis for many years. Daily contact with care for the opioid medication promoted adherence to the stimulant medication and allowed for monitoring of dose and tolerance. Settings where patients are in regular contact with care such as oral and injectable opioid agonist treatment clinics serve as a suitable location to integrate dextroamphetamine prescribing for patients that use illicit stimulants to reduce use and associated harms.

## Background

In the past decade overdose deaths have contributed a dramatic burden to population health in North America, such that adult life expectancy was declining between 2014 and 2017 in the United States (US) [1], and did not increase in Canada in 20162017 for the first time in over four decades [2]. Furthermore, a more recent and concerning pattern of polysubstance use has been observed in the form of co-occurring opioid and stimulant use. In British Columbia (BC), fentanyl (a synthetic opioid) was detected in 87% of all drug toxicity deaths where amphetamines were involved in 2017 [3] and in the US rates of fatal and non-fatal overdoses involving cocaine, both with and without opioids have been increasing [4]. The concurrent rising rates of stimulant use and opioid related overdose among people who use opioids in recent years in the US have been referred to as twin epidemics [5].

For people with opioid use disorder, oral opioid agonist treatment is effective at reducing illicit opioid use and retaining patients in treatment [6,8]. For those not well engaged in oral OAT, clinical trials in Canada and Europe support the effectiveness of injectable opioid agonist treatment (iOAT) with injectable hydromorphone (HDM: an opioid analgesic) [9, 10] or diacetylmorphine (DAM: pharmaceutical grade heroin) [11,14]. Despite the effectiveness of these medications at reducing illicit opioid use, illicit stimulant use remains high among people receiving treatment for opioid use disorder [15,17]. The concurrent use of cocaine among people with opioid use disorder is concerning, given it interferes with treatment outcomes in both oral and injectable OAT, for example predicting early treatment discontinuation and lower rates of treatment retention [15, 18, 19].

While effective and approved pharmacological treatments exist for opioid use disorder (opioid agonist treatments), there are no approved pharmacological treatments for stimulant use disorder in North America. Until recently, systematic reviews have not concluded the overall effectiveness of psychostimulant medications for cocaine [20] or amphetamine use disorders [21] given many of the included studies have small sample sizes, high dropout, and stringent outcome measures (e.g. urine positive for cocaine metabolites (i.e. abstinence)). A more recent meta-analysis however has concluded that prescription psychostimulants, particularly amphetamines such as dextroamphetamine, can have a clinically significant beneficial effect in promoting abstinence in the treatment of cocaine use disorder when robust doses are provided [22].

A number of studies have demonstrated promising evidence for the effectiveness of dextroamphetamine as a treatment for stimulant use disorder. For example, a randomized controlled trial (RCT) conducted in Australia found significant reductions in cocaine positive urine samples, self-reported craving, criminal activity, and severity of cocaine dependence in the dextroamphetamine arm but not in the placebo arm [23]. An RCT conducted among methadone patients in the US found that patients receiving 3060mg of dextroamphetamine had significant reductions in cocaine use compared to those receiving lower doses or placebo [24]. Results from studies of patients with comorbid opioid use disorder have been promising [22]. For example, among patients receiving injectable diacetylmorphine, a Dutch RCT concluded that dextroamphetamine was safe and effective for the treatment of cocaine-dependence, with significant reductions in urine positive for cocaine metabolites, self-report craving and days of cocaine use [25]. Secondary analysis found that the treatment supported significant improvements in health and social functioning [26]. Given the major public health concern of untreated psychostimulant use disorder, and lack of widely accepted pharmacological treatment, experts are increasingly advocating the need for implementation studies of treatment approaches such as dextroamphetamine prescribing [22, 27].

Following this evidence, physicians at the Providence Health Care Crosstown Clinic in Vancouver began to prescribe dextroamphetamine for patients with stimulant use disorder. In the context of COVID-19, dextroamphetamine prescribing has been offered together with flexible OAT dosing, and conversion from injectable to oral OAT has been offered to support patients who wish to limit clinic attendance to once per day to reduce potential COVID-19 exposure. This prescribing approach has attracted growing interest from other clinics offering OAT and iOAT in Canada and the US. Furthermore, in March 2020, in response to COVID-19, British Columbias provincial Ministry of Health developed guidelines for prescribing pharmaceutical alternatives to the toxic drug supply [28, 29]. Dextroamphetamine was listed as a medication that could be prescribed to people who use stimulants.

Despite mounting evidence of effectiveness from clinical trials, the prescribing of pharmacological treatments such as dextroamphetamine for the treatment of stimulant use disorder is not common practice in North America. This case report contributes a practical example of dextroamphetamine prescribing. Specifically, a case is presented of a patient who was prescribed dextroamphetamine for the treatment of cocaine use disorder at an iOAT clinic in Vancouver, BC. The clients history, engagement with the medication in the clinical setting, and outcomes are reported alongside a dosing protocol and medication information. To our knowledge, this is the first report of dextroamphetamine prescription offered for the treatment of stimulant use disorder among a patient receiving iOAT outside of a clinical trial. This novel information therefore holds significant potential educational value for care providers working with patients who use cocaine.

In the context of the previously outlined twin epidemics (i.e. rising rates of concurrent opioid and stimulant use), and the ongoing COVID-19 pandemic, people who use opioids and stimulants are at an elevated risk of overdose [30,32]. In the coming years (long-term), the evidence for a comprehensive range of prescription stimulants will continue to grow. In the short-term however, action is required. This case report can be used to inform clinical practice in the treatment of cocaine use disorder with dextroamphetamine in an effort to support patients for whom interventions and clinical reporting are greatly needed yet currently lacking.

## Case presentation

A 51-year-old male diagnosed with opioid and stimulant use disorder was receiving injectable opioid agonist treatment (iOAT) with hydromorphone (Sandoz Inc. 50mg/ml solution) at a community clinic in Vancouver. Prior to receiving iOAT he reported injecting illicit opioids and smoking crack cocaine daily for over two decades and had multiple prior oral OAT attempts. He was prescribed 200mg of hydromorphone three times per day (600mg total per day), since August of 2014 (with dose adjustments). He had trialled diacetylmorphine (pharmaceutical grade heroin) in February 2015 but preferred hydromorphone. Hydromorphone supported him to stop his illicit opioid injection however he reported ongoing daily crack cocaine smoking, on average 1015 rocks per day. He presented to his physician with the goal of reducing his crack cocaine use in January 2020.

His medical history included numerous conditions that were aggravated by smoking crack cocaine including diagnoses of hepatitis C, chronic obstructive pulmonary disease, and pulmonary hypertension. He had developed pulmonary embolism as a result of deep vein thrombosis moving to his lungs. The clot damaged the veins in both of his legs, and he developed associated chronic leg wounds. The ongoing use of crack cocaine contributed to poor wound healing. He reported engaging in drug dealing daily to support his use. He was motivated to reduce his crack cocaine use in order to: (1) limit his spending on cocaine, with a longer-term goal of saving money and; (2) to minimize its physical health burden. He reported that crack cocaine use helped him with remaining alert to accomplish daily tasks, and as such found it difficult to cut down on his crack cocaine use.

**Fig. 1:**
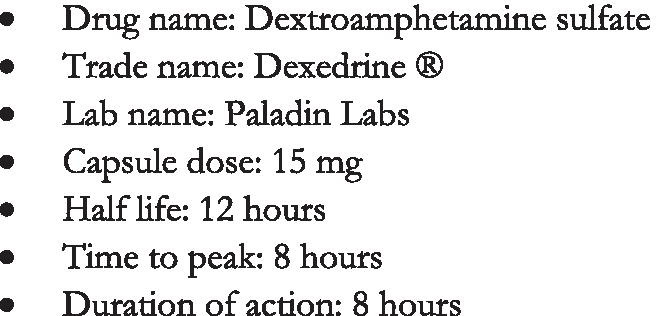
Dextroamphetamine sulfate drug description

**Fig. 2:**
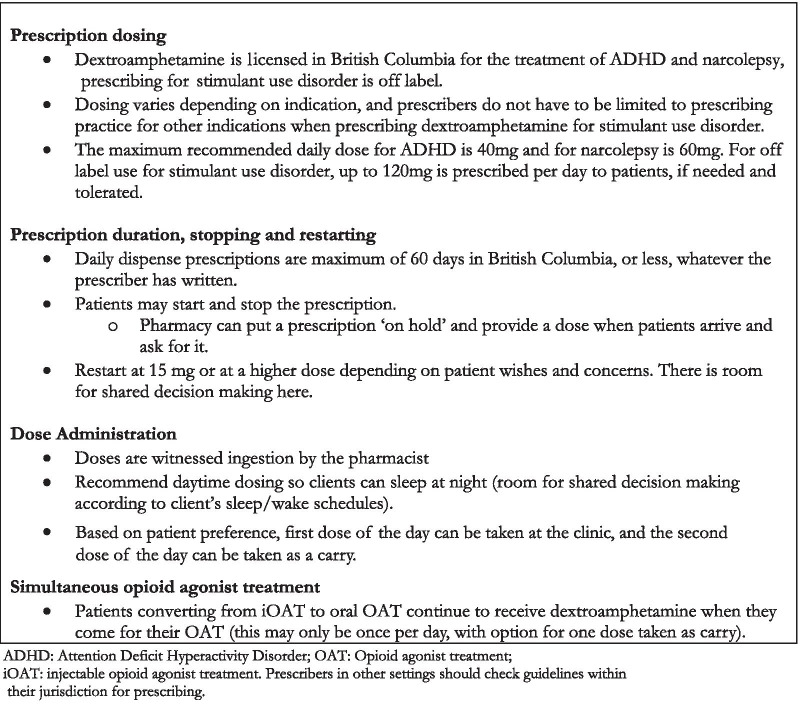
Dextroamphetamine sulfate administration procedures

A trial of sustained-release dextroamphetamine sulfate (Dexedrine ) (See Fig.1) was offered following the clinics dextroamphetamine dosing protocol (Table 1) and was taken at the clinics on-site pharmacy (See Fig.2). This was his first time receiving treatment for stimulant use disorder. Prescribing began on January 24th 2020 at 15mg twice per day, though the patient was advised to begin with one capsule and could increase to two if the dose was tolerated (i.e. no or mild side effects). On February 1st 2020, the patient reported tolerating 15mg of dextroamphetamine twice per day and did not report experiencing any adverse effects (e.g. sleeping problems, agitation, changes in appetite, etc.). He reported that his crack cocaine cravings were reduced, but not eliminated. The patient and physician decided together to increase the dose and a prescription for dextroamphetamine 30mg twice per day was written.

**Table 1:** Dextroamphetamine sulfate dosing: guidance and dose received by presented case

	Days 13		Days 47		Days 814		Days 1521		Days 2228	
	Clinic protocol (mg)	Case received (mg)	Clinic protocol (mg)	Case received (mg)	Clinic protocol (mg)	Case received (mg)	Clinic protocol (mg)	Case received (mg)	Clinic protocol (mg)	Case received (mg)
Daily Dose 1	15	15	15	15	30**	30	45***	30	60****	30
Daily Dose 2			15*	15	30**	30	45***	30	60****	30

This table reflects the dosing protocol followed at the clinic where the patient received this treatment. He tolerated and remained at 30mg twice per day

Tolerance is determined based on absence of adverse events, and patient reported therapeutic effect, in line with patient-reported goals

Start at 15mg (or lower if determined to be more suitable (i.e. 10mg capsules are available)). Increase incrementally as needed, every week or so. There is not usually any urgency and patients generally take some time to assess effect and tolerance. Table reflects general guidance, not all clients will need or want to reach 60mg twice per day

Patients are dispensed dextroamphetamine from the clinics on-site pharmacy. The pharmacy hours are 7:30am-5:30pm. Patients can take their first dextroamphetamine dose as early as 7:30am. The second dose can be taken anytime following the first dose, so long as four hours have passed between doses

*If dose 1 tolerated on days 13 **If dose 1, 2 tolerated on days 47

***If doses 1, 2 tolerated on days 814 ****If doses 1, 2 tolerated on days 1521

On February 23rd 2020, 4weeks after beginning the prescription, he reported less cravings and clinically significant reductions in use, from 1015 rocks daily to between 1 and 2 rocks on average two days per week. He reported having stopped his daily drug dealing as it was no longer required to support his crack cocaine use. He was meeting his self-identified therapeutic goals of reducing his crack cocaine use and reducing his spending on crack cocaine. The patient reported that dextroamphetamine helped him to feel energized, a feeling that he had previously been seeking from crack cocaine. Clinical improvements were witnessed in his abscesses and chronic wounds, which began to heal. In addition, care providers noted that he became more cognitively alert and was more engaged and interested in conversations. On May 18th 2020, the patient discontinued the medication, stating it was no longer required to maintain the reductions in cocaine use he had attained. Upon the most recent clinical visit on October 5th 2020, the patient reported having maintained the significant reductions in cocaine use, and the prescribing physician (SM) reported persistent clinical improvements in the patients physical health, cognition and social functioning. For example, the patient maintained his reduction of crack cocaine use, and experienced persistent improvements in mobility, and reduced edema (swelling) of the legs.

## Discussion and conclusions

We presented a case of dextroamphetamine prescribing for the treatment of stimulant use disorder in the context of a community clinics iOAT program. No adverse events were observed, consistent with the safety profile of this medication as demonstrated by prior clinical trials [25, 33,36]. For example, a prior clinical trial of dextroamphetamine prescribing in a heroin assisted treatment clinic, reported data on all adverse events (e.g. sleeping problems, agitation/irritability, gastrointestinal problems, changes in appetite, weight, dizziness, and craving). Sleeping problems were found to be the most commonly reported adverse event (reported by approximately one third of patients in the dextroamphetamine group). Heart rate, blood pressure, and body weight were monitored throughout the trial. While heart rate was found to increase from baseline to 12weeks among people receiving dextroamphetamine, electrocardiogram (ECG) data revealed no abnormalities, and no serious adverse events were observed [25]. In the present study setting, some patients reported an unpleasant sensation from the medication, which was usually vague and difficult to define. In these cases, the medication was discontinued with no adverse events, and the unpleasant sensations were resolved quickly.

The medication was delivered in an iOAT clinic, where adherence was promoted by the patients daily visits to the clinic for his opioid agonist medication. The observed benefits in terms of reduced use, coupled with the lack of adverse events, and daily contact with care for monitoring contributed to making this medication suitable for delivery in the iOAT care setting.

The patient was achieving his therapeutic goals of reducing cocaine use, and decreasing spending on cocaine while receiving dextroamphetamine. This finding suggests that future studies evaluating treatment effectiveness could benefit from extending measures of effectiveness. This could involve complementing measures of effectiveness from the perspective of the health care system (e.g. cocaine metabolites by urine drug screen (UDS) to measure abstinence) with other measures that are able to capture the progress that patients make while receiving this treatment. In the present study for example, following shared decision-making between the patient and prescriber, dextroamphetamine was prescribed with the objective of supporting reductions in (and not abstinence from) cocaine use. UDS to detect the presence of cocaine metabolites were not conducted and would not have captured the clinically significant reductions in his cocaine use the patient reported after decades of regular daily use.

A recent systematic review of the evidence for pharmacological treatment of stimulant use disorder [37] concluded that there exists a need for trials to focus on outcomes that can demonstrate treatment benefit without hinging on achievement of abstinence, for example measuring reductions in frequency of use or improvements in health-related outcomes. In the present case, the benefits of reducing cocaine use extended for example, to reducing engagement in drug dealing, and spending less money on cocaine. Prior studies have measured cocaine use using UDS alongside measures of cocaine craving, self-reported number of days of cocaine use [25] and physical and psychological health and social functioning [26].

Such an extension of outcomes of study beyond UDS, would represent an acknowledgement of the range of outcomes that contribute to patients well-being, beyond reductions in or abstinence from street drug use [37]. Such a shift in outcome measurement has relevance not just to the study of stimulant use, but to opioid use, and opioid agonist treatment (OAT) where the delivery of care has historically involved largely medicalized and routinized approaches (e.g. daily methadone dispensation), often devoid of social interaction or shared decision-making. Research on approaches to delivering OAT increasingly outline patients desires for more holistic approaches to treatment [38, 39]. For example, in Australia, the introduction of care for Hepatitis C (HCV) into OAT clinics was found to strengthen the therapeutic relationship between patients and clinicians and reduce the stigma associated with attending the clinic [40]. The incorporation of dextroamphetamine into iOAT care (alongside other health services such as HCV treatment), could similarly work to improve therapeutic relationships (e.g. between pharmacists and patients), and provides an important step toward providing comprehensive addiction care. Such comprehensive approaches can also begin to open clinicians to a growing awareness of the structural vulnerabilities their patients may face to accessing, and engaging in treatment services [41], and working with their patients to connect them to resources to best address these concurrent service needs (e.g. housing, education, social networks).

Furthermore while in this setting, dextroamphetamine was made available to all patients who used illicit stimulants and wanted to try it, it is important to acknowledge that all patients present to care with different motivations for and patterns of cocaine use. For example, this case suggests that the alignment between the illicit stimulant and prescribed stimulant in terms of perceived effect might be an important determinant of treatment benefit. The patient reported using cocaine to feel energized and help with remaining alert. When dextroamphetamine offered him the feeling of being energized he had been seeking from cocaine he was able to reduce his cocaine use. In settings where dextroamphetamine may be considered as a treatment option, patients motivations for illicit stimulant use (e.g. seeking a rush, etc.) are important considerations to be discussed by the prescriber and patient, along with expectations of the medication and its possible benefits (e.g. improved focus, reduced craving, etc.) and side-effects (e.g. interfering with sleep, agitation, etc.).

Dextroamphetamine may not be suited to all patients with stimulant use disorder. Efforts to expand treatment services will therefore be important to move toward a system of care that incorporates a comprehensive range of treatment options to meet patients diverse needs [27]. Further clinical trials and implementation studies can be conducted to extend the evidence. For example, the presented case chose to discontinue treatment after approximately 16weeks, and prior RCTs have been short in duration (i.e. 1012weeks). While OAT is known to serve as a long-term treatment for opioid use disorder, the optimal duration of treatment with dextroamphetamine is not well understood. Longer-term studies could be conducted to examine particular treatment outcomes over time. Furthermore, the case received up to 60mg of dextroamphetamine per day. Nevertheless, the clinics dosing protocol allowed doses of up to 120mg/day in order to meet patient preferences. This was made possible by prescribing in a clinical treatment program, rather than in the context of a strict clinical trial, where the dosing protocol would have been determined a priori, and would not have been adapted based on patient preferences. This increased maximum daily dose was in line with recommendations from a recent meta-analysis which concluded that robust doses (i.e. 60mg or more/day for prescription amphetamines) are required in order to achieve positive outcomes [22]. As such, future clinical trials and implementation studies will have the greatest chances of success where robust doses are provided.

Furthermore, beyond dextroamphetamine, other stimulants show modest effects in the treatment of stimulant use disorder, including methylphenidate (Ritalin) [21, 37, 42] and other treatments are being tested within specific populations. The prevalence of concurrent Attention Deficit Hyperactivity Disorder (ADHD) for example is high among people who use illicit stimulants and prescribed stimulants like extended-release mixed amphetamine salts (Adderall ) can improve symptoms of both cocaine use disorder and ADHD [43]. Given dextroamphetamine is indicated for the treatment of ADHD in Canada, it could be prescribed to patients with co-occurring ADHD and stimulant use disorder, with potentially beneficial effects on the symptoms of both conditions. Dextroamphetamine has also been trialed with positive outcomes among people who use methamphetamine [33, 34, 37]. A recent systematic review further suggested that dextroamphetamine and methylphenidate show the most promise as treatments for stimulant use disorder in people that use methamphetamine, and that future studies should focus on these treatments [37].

Expanding access to dextroamphetamine and other evidence-based treatments requires a systems approach that can adapt to overcome potential barriers and integrate evidence-based interventions as they are available. In the US psychostimulants have been tested in RCTs, and given the rising rates of cocaine and methamphetamine use across the country, efforts are mounting to increase the accessibility of these medications [44, 45]. Leading addictions experts in North America including Dr. Nora Volkow of National Institute on Drug Abuse continue to highlight the need for medications to strengthen the response to illicit stimulant use [46].

In Canada dextroamphetamine is licensed for the treatment of ADHD and narcolepsy and can be prescribed off-label for stimulant use disorder. Logistical and operational costs and concerns can be minimized by offering this medication in settings where patients are already in regular (often daily) contact with care (e.g. community clinics). Such settings are amenable to high rates of adherence, given patients are motivated to attend care for other services [25]. The presented case received dextroamphetamine at his iOAT clinics on-site pharmacy, a site he visited three times daily for iOAT. This facilitated treatment adherence given the opioid medication provided an additional motivation for clinic visits. Adherence was further promoted by the on-site pharmacist who offered medication reminders and education. In the scaling up of this medication, pharmacists can play a lead role by dispensing medication, monitoring side-effects, ensuring proper administration (e.g. enough hours between doses), and offering medication reminders [47].

This case report has a number of important limitations to consider. First, UDS are often captured to complement self-reported patient outcomes in clinical case reports. In the present case, the goal of treatment at the time of prescribing, as discussed by the patient and provider was to reduce cocaine use, and not to achieve abstinence. Therefore, UDS to detect the presence of cocaine metabolites (already self-reported by the patient) did not serve a clinical purpose and were not recorded. The presented outcomes therefore are derived from clinical records and patient self-report. Second, the case presented with a particular profile of long-term concurrent use of opioids and stimulants, and was receiving dextroamphetamine in a specific clinical setting of iOAT. Future studies may wish to examine the suitability of this treatment to other settings such as community clinics and pharmacies. Reporting of case findings was strengthened by regular communication between the patient and prescribing physician (SM) which fostered involvement of the patient in the case presentation, to ensure an accurate depiction of his experience.

This case report presents a treatment approach that can be used to support patients to reduce their use of cocaine, particularly in structured care setting such as oral or injectable OAT clinics. Dextroamphetamine has been adopted in some clinical care settings in Canada, serving a minority of the many patients that could benefit from this treatment. As efforts continue to advance access to this medication, patients motivations for use and self-identified treatment goals can be centered in care plans to support the achievement of positive outcomes.

## Data Availability

Data sharing is not applicable to this article as no datasets were generated or analysed during the current study.

## Abbreviations

ADHD: Attention deficit hyperactivity disorder
iOAT: Injectable opioid agonist treatment
OAT: Opioid agonist treatment
OUD: Opioid use disorder
